# Associations of eHealth Literacy With Health Services Utilization Among College Students: Cross-Sectional Study

**DOI:** 10.2196/jmir.8897

**Published:** 2018-10-25

**Authors:** Yi Fang Luo, Shu Ching Yang, An-Sing Chen, Chia-Hsun Chiang

**Affiliations:** 1 Institute of Education National Sun Yat-sen University Kaohsiung Taiwan; 2 Department of Finance National Chung Cheng University Chiayi Taiwan

**Keywords:** eHealth, health literacy, health services, student, utilization

## Abstract

**Background:**

Electronic health (eHealth) literacy has become an important topic in health fields. Studies have found that individuals with higher eHealth literacy are more likely to use preventive care services and to have effective interactions with their physicians. In addition, previous studies have revealed a gender difference in the utilization of physician access and outpatient services. Nevertheless, few studies have explored the effect of the three levels of eHealth literacy (functional, interactive, and critical levels) on the four aspects of health services utilization (type, site, purpose, and time interval). It is unclear whether the associations between these three levels of eHealth literacy and the four aspects of health services utilization among college students are positive or negative.

**Objective:**

The objective of this study was to investigate the associations among gender, eHealth literacy, and health services utilization.

**Methods:**

We used the eHealth Literacy Scale, a 12-item instrument designed to measure college students’ functional, interactive, and critical eHealth literacy, and the Health Services Utilization Scale, which is a 10-item instrument developed to measure the four aspects of health services utilization by college students. A nationally representative sample of 489 college students in Taiwan was surveyed. We conducted multiple regression analysis to examine the associations among gender, eHealth literacy, and health services utilization.

**Results:**

The study found that being female was negatively related to the purpose aspect of health services utilization (*t*_487_=−2.85, *P*<.01). However, the *R^2^* value of gender on the purpose aspect was low enough to be ignored. Critical (*t*_484_=2.98-4.23, *P*<.01) and interactive eHealth literacy (*t*_484_=2.43-2.89, *P*<.05) were related to three aspects of the health services utilization, and functional eHealth literacy was related to the purpose aspect (*t*_484_=−4.99, *P*<.001).

**Conclusions:**

This study showed that Taiwanese college students with interactive eHealth literacy were more likely to have a higher rate of outpatient care use. Moreover, Taiwanese college students with critical eHealth literacy were more likely to make full use of health services than those with functional eHealth literacy. Finally, the educated and age-restricted sample may attenuate gender disparities in health services utilization among Taiwanese college students.

## Introduction

### Health Literacy and eHealth Literacy

In the past, individuals were likely to obtain health information from written sources such as books, magazines, newspapers, or brochures. The advent of the internet has drastically changed health information, and the internet is widely used to obtain this information. However, the skills needed to collect information through the internet differ from those needed to collect information from books and leaflets. People need to not only be health literate but also have capabilities, resources, and motivation to find, understand, and appraise health information when using digital services and technology [1]. Therefore, to obtain a complete overview of people’s skills in obtaining and using health information, it is more necessary to measure electronic health (eHealth) literacy than to measure health literacy [2].

eHealth literacy is an extension of the health literacy concept. Health literacy refers to an individual’s ability to understand health care information and to make appropriate decisions [3]. eHealth literacy, which consists of skills related to health literacy and digital literacy [1], is defined as the ability to seek, find, understand, and appraise health information from electronic sources and to apply the knowledge gained to address or solve health problems [4]. Based on Nutbeam’s concept [5], eHealth literacy includes functional, interactive, and critical levels. At the most basic level, functional eHealth literacy refers to basic reading and writing skills and basic knowledge of health conditions and health systems. Interactive eHealth literacy refers to communicative and social skills that can be used to abstract information and derive meaning from different forms of communication. The highest level of critical eHealth literacy builds on functional and interactive literacy and involves the most advanced cognitive skills that can be applied to critically analyze information, discern the quality of health websites, and use good information to make informed decisions about health [6,7].

eHealth literacy has become an important topic in the health fields. Studies have found that individuals with high eHealth literacy are more likely to adopt healthy behaviors [6,8] and demonstrate better health responsibility and self-actualization [7]. Recently, access to health services and the relational dynamics between patients and health care providers have been transformed by Web-based health information-seeking behaviors [9] and eHealth literacy [10]. Therefore, the aim of this study is to examine the extent to which functional, interactive, and critical eHealth literacy are associated with college students’ utilization of health services.

### Health Services Utilization

**Figure 1:**
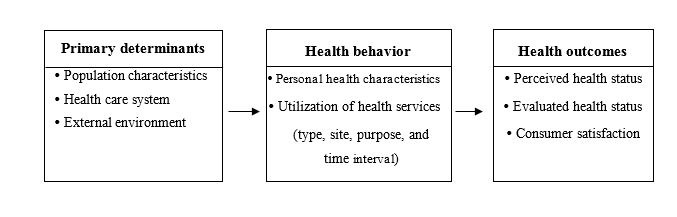
Andersen model of health services utilization.

Andersen [11] suggests four relevant aspects of the utilization of health services, each reflecting different aspects of the care-seeking process (Figure 1). The type of utilization indicates what kind of service is obtained and who provides it (eg, hospital, physician, dentist, or pharmacist). The site of the medical care encounter is the place where the care is received (eg, physician’s office, hospital clinic, or emergency room). The purpose of the visit signifies the reasons for care-seeking such as a need for preventive, illness-related, or custodial care. The time interval for a visit can be represented by contact, volume, or continuity measures [12].

Gender has been identified as a predisposing factor of health services utilization [13-16]. As increasing numbers of studies investigate the relationship between gender and health services utilization in different contexts, important inconsistencies have been reported. For example, some studies have found that females are more likely than males to visit physicians [17] and tend to use more outpatient services [18]. Studies have also found that females perceive a greater need for mental health treatment [19] and have a higher tendency to use all services, except informal providers, than males [20]. However, other studies have shown that females suffering from illness report seeking health care less frequently than men [21]. Females were found to be more likely to report discrimination in health care and less likely to receive preventive health services [22]. Furthermore, other studies have found no statistically significant evidence of a relationship [23,24]. College students are an educated and transition-age population group. They are in the upper part of the distribution in terms of their resources. Previous studies have indicated that the combination of education [25] and women’s autonomy [26] appear to attenuate gender disparities in health services utilization. Therefore, we want to investigate whether there are gender differences in the four aspects of health services utilization among college students.

### Relationship Between eHealth Literacy and the Utilization of Health Services

Individuals with limited health literacy are at an increased risk of poorer health outcomes. Systematic reviews have demonstrated that low health literacy is consistently associated with more hospitalizations, greater use of emergency care, and lower receipt of mammography screening and influenza vaccine [27]. In contrast, higher health literacy supports effective involvement in care processes. Studies have shown that individuals with adequate health literacy are more likely to be satisfied with care [28] and use preventive health services [29].

Web-based health information-seeking behaviors and eHealth literacy are transforming the physician-patient relationship and health services [9,10]. People who use eHealth resources (eg, health websites) are more likely to be well prepared for clinical visits, ask more related questions, learn more about their health care, and take actions to enhance their health than those who do not [30]. Studies have shown that internet use is associated with increased use of health care services and better-perceived outcomes of internet use for health purposes [31], including the decision to use health services and the way individuals communicate with physicians and request referrals to specialist care [32]. In addition, studies have shown that individuals with higher eHealth literacy are more likely to have knowledge of and a screening history for colorectal cancer [33] and more effective interactions with their physicians [34]. Moreover, some studies have found a set of indirect effect paths, via health information seeking and empowerment, showing a positive relationship between eHealth literacy and health care utilization [35]. However, these studies did not examine the effect of the three levels of eHealth literacy on the four aspects of medical services usage. Therefore, the research question examined for this study is as follows: Are the associations between the three levels of eHealth literacy and the four aspects of health services utilization among college students positive or negative?

## Methods

### Participants

#### Pretest Sample

Pretesting can help authors identify questions that do not make sense to participants as well as problems with questionnaires that might lead to biased answers. Thus, pretesting was conducted to develop and test the adequacy of the research instrument designed by the authors. Confirmatory factor analysis (CFA) was used to assess the reliability of the survey instrument. A reasonable sample size for a simple CFA model is approximately N=100-150 [36]. For this study, a purposive sample of 240 college students was drawn from 1 comprehensive university in Taiwan. Each participant was mailed a questionnaire, and 193 usable (completed) questionnaires were returned, resulting in an effective response rate of 80.4% (193/240).

#### Formal Study Sample

This cross-sectional study was conducted in Taiwan. We recruited 600 college students from 9 schools to participate in the survey. Teachers in selected colleges were contacted to request their assistance in the questionnaire distribution. Of the 529 recovered surveys, 40 invalid surveys (respondents had not completed the entire survey or gave invalid responses) and 489 valid surveys (92.4%, 489/529) were retained. The number of valid surveys was >350 [37]; thus, we deleted the invalid surveys. Among these 489 valid respondents (183 male and 306 female), 28.0% (137/489) studied in the northern region, 22.3% (109/489) studied in the central region, and 49.7% (243/489) studied in the southern region of Taiwan. The participants’ mean (SD) age was 21.51 (4.11) years. Of the 489 participants, 85.3% (417/489) were aged <22 years.

### Survey Instrument

#### eHealth Literacy Scale

eHealth Literacy was assessed by Chiang et al’s eHealth Literacy Scale (eHLS) [8]. The validity and reliability of Chiang et al’s eHLS were tested using item analyses, exploratory factor analysis, and CFA, which revealed that the eHLS is a reliable and validated measure of functional, interactive, and critical eHealth literacy for Taiwan college students. The 12-item eHLS assesses functional (3 items, eg, the Web-based health information is too difficult to understand), interactive (4 items, eg, paying attention to Web-based health information), and critical (5 items, eg, evaluating the effectiveness and reliability of Web-based health information) eHealth literacy. Responses were given on a 5-point Likert scale ranging from 5 (total agreement) to 1 (total disagreement). Mean scores for the eHLS were calculated by summing the item scores divided by the total number of items, resulting in a score ranging from 1 (lower eHealth literacy) to 5 (higher eHealth literacy).

Amos 6.0 CFA was used to examine the best measurement model. An analysis was conducted with Amos using maximum likelihood estimation. The factor loading of the 12-item eHLS ranged from 0.61 to 0.86. The individual item reliability of the 12-item eHLS ranged from 0.38 to 0.74.

The composite reliability ranged from 0.80 to 0.91, and the average variance extracted for each level ranged from 0.57 to 0.66. The cutoff values of CFA included a chi-square/degree of freedom value of <3, a root mean square residual (RMR) value of <0.50, a standardized root mean square residual value (SRMR) of <0.50, a root mean square error of approximation (RMSEA) value of <0.08, a goodness of fit index (GFI) value of >0.90, an adjusted GFI (AGFI) value of >0.90, a normed fit index (NFI) value of >0.90, a Tucker-Lewis index (TLI) value of >0.90, a relative fit index (RFI) value of >0.90, an incremental fit index (IFI) value of >0.90, a comparative fit index (CFI) value of >0.90, a parsimonious goodness of fit index (PGFI) value of >0.50, a parsimonious normed fit index (PNFI) value of >0.50, and a parsimonious comparative fit index (PCFI) value of >0.50 [38,39]. We found that the current data adequately fit the eHLS model, which was divided into 3 levels (total 12 items). We used Amos to conduct CFA, and a review of the fit indices revealed a chi-square/degree of freedom value of 1.68, an RMR value of 0.02, an SRMR value of 0.40, an RMSEA value of 0.06, a GFI value of 0.93, an AGFI value of 0.90, an NFI value of 0.94, a TLI value of 0.97, an RFI value of 0.92, an IFI value of 0.97, a CFI value of 0.97, a PGFI value of 0.61, a PNFI value of 0.72, and a PCFI value of 0.75. Furthermore, the chi-square test was significant (χ^2^_51_=85.50, *P*=.002). However, the chi-square statistic is, in essence, a statistical significance test that is sensitive to sample size, which means that this statistic nearly always rejects the model when large samples are used [38,40].

#### Health Services Utilization Scale

We developed the Health Services Utilization Scale (HSUS) following a thorough review of the literature [12,23] and applying the process of concept clarification. The content validity of the HSUS was pretested by three specialist professors. It was also pretested with 5 college students. It contains the following four dimensions (Multimedia Appendix 1):

1. Type: The tendency to make good use of various kinds of health care service organizations (3 items, eg, receiving various forms of medical treatment such as Chinese medicine, Western medicine, and dental services).
2. Site: The tendency to make good use of a multitiered health care system (2 items, eg, choosing a suitable site for medical services such as a physician’s office, hospital clinic, or emergency room).
3. Purpose: The ability to seek medical advice based on different needs (3 items, eg, visiting a physician for preventive care).
4. Time interval: The frequency of medical use (2 items, eg, obtaining a second opinion from another physician).

The items were answered using a 5-point Likert scale with scores ranging from 1 (never) to 5 (always). High scores in the respective aspects indicated a greater likelihood of the respondents making good use of various kinds of health care services and a multitiered health care system, seeking medical advice based on different needs, and having a greater frequency and ratio of outpatient care use.

The current data had a good fit with the model, which was divided into 4 aspects (10 total items). We used Amos to conduct CFA and found that the factor loading of the 10-item HSUS ranged from 0.66 to 0.82. The individual item reliability of the 10-item HSUS ranged from 0.43 to 0.68. The composite reliability ranged from 0.72 to 0.77, and the average variance extracted for each aspect ranged from 0.51 to 0.58. A review of the fit indices revealed a chi-square/degree of freedom value of 2.12, an RMR value of 0.05, an SRMR value of 0.50, an RMSEA value of 0.08, a GFI value of 0.94, an AGFI value of 0.89, an NFI value of 0.91, a TLI value of 0.92, an RFI value of 0.90, an IFI value of 0.95, a CFI value of 0.95, a PGFI value of 0.50, a PNFI value of 0.59, and a PCFI value of 0.61. Furthermore, the chi-square test was significant (χ^2^_29_=61.42, *P*<.001). According to the cutoff values of CFA [38,39], the indicators demonstrated a good fit for the measurement model.

### Data Analysis

First, peer review was used to confirm the content validity of the HSUS. Second, we used Amos 6.0 (IBM Corp, Armonk, NY, USA) to perform CFA to identify the best measurement models for the eHLS and HSUS. Finally, SPSS 18.0 (IBM Corp, Armonk, NY, USA) was used to conduct hierarchical multiple regression analysis. We performed four hierarchical multiple regression analyses to examine the explanatory power of the four aspects of health services utilization. We determined the order in which the variables were entered into the model based on logical or theoretical considerations. Thus, gender was entered in step 1, and the three levels of eHealth literacy were entered in step 2.

### Ethical Considerations

The study was reviewed and approved by the Institute of Education at the National Sun Yat-Sen University, Taiwan. The study adopted an anonymous questionnaire, in line with the government’s institutional review board rules for exempt review. The questionnaire instructions informed the participants of the research purpose and confidentiality and indicated that they had the right to refuse to participate at any time. The participants received the questionnaire and gifts at the same time; even if a participant decided to drop out of the investigation, he or she still received the gifts (a pen and an L-folder). This approach was intended to be fair to each participant, to avoid the impact of gift incentives on the participants, and to provide compensation for the participants.

## Results

### Descriptive Statistics of eHealth Literacy and Health Services Utilization

Among all participants, the mean score of functional eHealth literacy was 3.66 (SD 0.70), of interactive eHealth literacy was 3.67 (SD 0.67), and of critical eHealth literacy was 3.65 (SD 0.69), indicating that college students had medium or above levels of eHealth literacy.

In HSUS, the mean score of the type aspect was 3.36 (SD 0.75), of site was 3.32 (SD 0.77), of purpose was 3.70 (SD 0.81), and of time interval was 2.37 (SD 0.80). This result indicated that college students had a certain degree of ability to make good use of various kinds of health care services and a multitiered health care system and to receive health care services based on different needs, although they did not frequently use outpatient care.

### Analysis of Gender and Health Services Utilization

In the analysis of Model 1 (see Multimedia Appendix 2), male and female groups were transformed into dummy variables. We used the male group as the reference group. Multimedia Appendix 2 indicates that membership in the female group was negatively related to the purpose aspect of health services utilization, yielding a low explanatory power of 2%. However, the *R^2^* value is very low to nil.

### Analysis of Gender, eHealth Literacy, and Health Services Utilization

When comparing Models 1 and 2 (see Multimedia Appendix 3), the level of explanatory power for the 4 health services utilization aspects increases by 6%-16%. Moreover, Multimedia Appendix 3 shows that when controlling for gender, interactive and critical eHealth literacy are both positively related to three aspects of health services utilization. Functional eHealth literacy is negatively related to the time interval aspect.

## Discussion

### Principal Findings

This study found no gender difference in health services utilization among Taiwanese college students. There was a statistically significant correlation between eHealth literacy and health services utilization. Finally, among the three eHealth literacy levels, functional eHealth literacy showed the lowest correlation to health services utilization.

The study found no significant gender differences in health utilization. Notably, the *R*^*2*^ value of gender on the purpose aspect was low enough to be ignored. The association between gender and service utilization has been inconsistent across studies. Some studies have found gender differences in health services utilization [17-22], whereas others have found no statistically significant evidence of a relationship [23,24]. The study sample was educated and age-restricted, specifically, young people and college students, who are in the upper part of the distribution in terms of resources. To some extent, education can improve the ability of individuals to increase their knowledge of modern health care, make decisions regarding their own health, and realize the benefits of using health services. There is ample evidence that education is a significant factor of service utilization [41,42]. Thus, the educated and age-restricted sample may attenuate gender disparities in health services utilization among Taiwanese college students.

Consistent with previous studies [43-45], this study found that functional eHealth literacy was negatively related to the time interval aspect. Functional literacy refers to basic reading and writing skills and the ability to understand and use health information [5]. Poor functional health literacy hinders individuals’ full understanding of personal health, disease, and treatment [46]. Individuals with low functional literacy may receive ineffective care because they do not comprehend medical care directions [44], and they may have insufficient problem-solving abilities or be unlikely to change their behavior based on new information [45]. In this study, the shorter time interval indicates the higher frequency of medical use. Taiwan’s National Health Insurance (NHI) system uses a third-party payer mechanism to cover medical expenses, making it extremely convenient and inexpensive for individuals to obtain care. Short or negligible waiting times stand out as hallmarks of the NHI system. However, the NHI system has also resulted in a surge in the “volume” of care provided and a sharp rise in medical expenses. Under the convenient NHI system, Taiwanese college students with inadequate functional eHealth literacy may visit physicians frequently in order to achieve the same therapeutic goal.

This study found that interactive eHealth literacy was positively related to the type, site, and time interval aspects of health services utilization. Under Taiwan’s NHI system, the insured can visit any NHI-contracted hospital, clinic, pharmacy, or medical laboratory for access to health care. In recent years, the NHI Administration inaugurated a new copayment fee schedule and referral to a multitiered health care system to encourage patients to seek treatment for basic ailments at local clinics and then obtain referrals to regional hospitals or medical centers if more advanced treatment or tests are necessary. Interactive literacy involves more advanced cognitive and literacy skills that can be used to actively participate in everyday activities and to transfer the information received to one’s own situation [5]. Interactive health literacy can augment one’s ability to act independently, enhance motivation, and improve self-confidence [47], thus enabling Taiwanese college students to choose suitable types of and sites for health care services. It is worth noting that interactive health communication has the potential to improve the practice of medicine and the structure of health care systems but may also cause harm [48]. Studies have found that >15% of internet users said they felt overwhelmed and confused by the amount of information they found on the web. Another 54% of internet users said that the information they found led them to ask their physician a new question or to visit another physician to obtain a second opinion [49]. Thus, Taiwanese college students with high interactive literacy may visit physicians frequently in order to clarify or confirm confusing Web-based information and their illness condition.

This study found that critical eHealth literacy was positively related to the type, site, and purpose aspects of health services utilization. Taiwan’s NHI system provides not only outpatient care service but also integrated preventive health care service. The goals of integrated preventive health care service include increasing the utilization of preventive care services, the early detection and intervention of high-risk and preclinical cases, and integrating the three levels of prevention, namely, health promotion and education, early detection and treatment, and the medical care system. Critical literacy involves the most advanced cognitive skills, which can be applied to critically analyze information and to use this information to exert greater control over life events and situations [5]. Critical literacy allows individuals to evaluate health issues and recognize risks and benefits as well as to advocate for themselves [50], thus enabling Taiwanese college students to choose suitable types of and sites for health care services. It also increased the utilization of health care services based on different needs (eg, preventive care, custodial care, and illness-related care).

Finally, the study found that functional eHealth literacy showed the lowest correlation to health services utilization among the three eHealth literacy levels. Functional health literacy rarely involves interactive communication and does not provide individuals with the skills to take action in their own community [51]. It is not sufficient for individuals to obtain health information; they must further evaluate and use it to make decisions about their health. Thus, in the process of functional eHealth literacy, individuals do not engage as deeply with issues as they do in the processes of interactive and critical eHealth literacy. Another explanation is that age and education play considerable roles in functional eHealth literacy. Many studies have shown that adults with functional health literacy are more likely to be younger and have higher levels of education [52,53]. The participants (average age, 21.51 years) in this study were higher education students, which facilitates functional eHealth literacy. Functional eHealth literacy showed the lowest correlation to health services utilization among the three eHealth literacy levels in this educated and age-restricted sample. Previous studies have also found that functional eHealth literacy has the lowest correlation to health behaviors and health-promoting lifestyles among college students [6-8].

### Limitations

The study sample was educated and age-restricted. Population characteristics are noted as direct factors of health services utilization [11,13]. However, this study did not collect information on participant variables such as socioeconomic status or marital status. Thus, the findings should not be overgeneralized and should be interpreted in light of the sample’s homogeneity. Additionally, our measure of health services utilization was not validated in the published literature. Future studies should develop and construct an HSUS. Moreover, this study found that functional eHealth literacy was related only to the time interval aspect of health services utilization. Other mediating or confounding variables may exist that should be taken into consideration such as health information seeking and increased empowerment through information seeking [35].

### Conclusions

The establishment of a link between the three levels of eHealth literacy and the four aspects of health services utilization is the unique contribution of this study. Over the past decade, few attempts have been made to examine other components of eHealth literacy, such as the ability to extract and critically analyze information and use it to make decisions, which should be part of interactive and critical eHealth literacy. Notably, this study found that functional and interactive eHealth literacy were related to the time interval aspect. This finding has important implications for health care providers, who should provide patients with information leaflets to help them understand their illness and, thereby, reduce the waste of medical resources. Given that no significant gender differences in health services utilization were identified, the educated and age-restricted sample appeared to attenuate gender disparities in health services utilization. The associations among gender, age, education, and health services utilization are worth examining in further studies.

## Appendix

Multimedia Appendix 1 Health Services Utilization Scale.

Multimedia Appendix 2 Hierarchical multiple regression analysis of four aspects of health services utilization (Model 1).

Multimedia Appendix 3 Hierarchical multiple regression analysis of four aspects of health services utilization (Model 2).

## Abbreviations

AGFI: adjusted goodness of fit index
CFA: confirmatory factor analysis
CFI: comparative fit index
eHealth: electronic health
eHLS: eHealth Literacy Scale
GFI: goodness of fit index
HSUS: Health Services Utilization Scale
IFI: incremental fit index
NFI: normed fit index
NHI: National Health Insurance
PCFI: parsimonious comparative fit index
PGFI: parsimonious goodness of fit index
PNFI: parsimonious normed fit index
RFI: relative fit index
RMR: root mean square residual
RMSEA: root mean square error of approximation
SRMR: standardized root mean square residual
TLI: Tucker-Lewis index
