# Supplementary material for: Associations of eHealth Literacy With Health Services Utilization Among College Students: Cross-Sectional Study
Source: J Med Internet Res. 2018 Oct 25;20(10):e283. doi: 10.2196/jmir.8897 (PMC6231732; doi:10.2196/jmir.8897)
Supplement: Multimedia Appendix 1 [file jmir_v20i10e283_app1.pdf]

aspect	item
type	1. Receiving various forms of medical treatment, such as Chinese medicine, Western medicine, and dental services.
	2. Receiving medical services at medical institutions such as hospitals, clinics and pharmacies.
	3. Choosing suitable clinic for outpatient care.
site	4. Seeing a doctor at a primary-level clinic near my home.
	5. Choosing a suitable site for medical services, such as physician’s office, hospital clinic, or emergency room.
purpose	6. Visiting a physician for preventive care.
	7. Visiting a physician for illness-related care.
	8. Visiting a physician for custodial care.
time interval	9. Obtaining a second opinion from another physician.
	10. The frequency of outpatient care use.
