# Supplementary material for: Associations of eHealth Literacy With Health Services Utilization Among College Students: Cross-Sectional Study
Source: J Med Internet Res. 2018 Oct 25;20(10):e283. doi: 10.2196/jmir.8897 (PMC6231732; doi:10.2196/jmir.8897)
Supplement: Multimedia Appendix 2 [file jmir_v20i10e283_app2.pdf]

Variable		Type				Site				Purpose				Time interval			
		B	Beta	T_{487}	*P* value	B	Beta	*t*	*P* value	B	Beta	*t*	*P* value	B	Beta	*t*	*P* value
**Model 1**																	
	Female	−.04	−.03	−.60	.55	−.07	−.04	−.95	.34	−.20	−.13	−2.85	.005	−.11	−.07	−1.52	.13
		R=.03, R^2=.00				R=.04, R^2=.00				R=.13, R^2=.02				R=.07, R^2=.01			
		$F_{1,487}$=.36				$F_{1,487}$=.91				$F_{1,487,}$=8.11				$F_{1,487}$=2.30			

[a]ΔR^2: R-squared change.
