# Supplementary material for: Associations of eHealth Literacy With Health Services Utilization Among College Students: Cross-Sectional Study
Source: J Med Internet Res. 2018 Oct 25;20(10):e283. doi: 10.2196/jmir.8897 (PMC6231732; doi:10.2196/jmir.8897)
Supplement: Multimedia Appendix 3 [file jmir_v20i10e283_app3.pdf]

| Variable | Type | | | | Site | | | | Purpose | | | | Time interval | | | |
|---|---|---|---|---|---|---|---|---|---|---|---|---|---|---|---|---|
| | B | Beta | $T_{484}$ | *P* value | B | Beta | *t* | *P* value | B | Beta | *t* | *P* value | B | Beta | *t* | *P* value |
| | | | | | | | | | | | | | | | | |
| **Model 2** | | | | | | | | | | | | | | | | |
| Female | −.02 | −.01 | −.26 | .79 | −.03 | −.02 | −.48 | .63 | −.18 | −.11 | −2.63 | .009 | −.14 | −.09 | −1.94 | .13 |
| **eHealth literacy** | | | | | | | | | | | | | | | | |
| Functional | −.01 | −.01 | −.28 | .78 | .01 | .01 | .26 | .79 | −.01 | −.01 | −.28 | .78 | −.26 | −.23 | −4.99 | <.001 |
| Interactive | .17 | .15 | 2.43 | .016 | .21 | .17 | 2.89 | .004 | .06 | .06 | .88 | .38 | .21 | .18 | 2.84 | .005 |
| Critical | .20 | .19 | 2.98 | .003 | .30 | .25 | 4.23 | <.001 | .29 | .26 | 4.21 | <.001 | .00 | .00 | .04 | .97 |
| | $R$=.31, $R^2$=.10 [a]$\Delta R^2$=.10 | | | | $R$=.40, $R^2$=.16 $\Delta R^2$=.16 | | | | $R$=.33, $R^2$=.11 $\Delta R^2$=.09 | | | | $R$=.26, $R^2$=.07 $\Delta R^2$=.06 | | | |
| | $F_{4,484}$=17.02 | | | | $F_{4,484}$=30.86 | | | | $F_{4,484}$=16.14 | | | | $F_{4,484}$=10.83 | | | |

[a]$\Delta R^2$: R-squared change.
